# Evaluation of PCR *pncA*-restriction fragment length polymorphism and PCR amplification of genomic regions of difference for the identification of *M. bovis* strains in lymph nodes cultures

**DOI:** 10.4314/ahs.v21i3.4

**Published:** 2021-09

**Authors:** Imen Bouzouita, Henda Draoui, Samia Mahdhi, Leila Essalah, Leila Slim Saidi

**Affiliations:** 1 National Reference Laboratory for mycobacteria, A. Mami pneumology hospital, Ariana, Tunisia; 2 University of Tunis El Manar, Faculty of Mathematical, Physical and Natural Sciences of Tunis, Tunis, Tunisia; 3 University of Monastir, Faculty of pharmacy, Monastir, Tunisia

**Keywords:** GenoType MTBC, lymph nodes, *Mycobacterium bovis*, PCR pncA-RFLP, RD-PCR

## Abstract

**Background:**

A rapid accurate identification of *Mycobacterium bovis* is essential for surveillance purposes.

**Objectives:**

A PCR *pncA*-Restriction Fragment Length Polymorphism (RFLP) and a multiplex PCR based on the detection of 3 regions of difference (RD-PCR): RD9, RD4 and RD1 were evaluated for the identification of *M. bovis* in lymph nodes cultures, in Tunisia, during 2013–2015.

**Methods:**

Eighty-two *M. tuberculosis* complex strains were identified using the biochemical tests, GenoType MTBC assay, PCR *pncA*-RFLP and RD-PCR.

**Results:**

The PCR *pncA*-RFLP showed that 54 *M. bovis* strains, identified by GenoType MTBC, had a mutation at position 169 of pncA gene. Twenty-eight strains did not show any mutation at this position 27 *M. tuberculosis* isolates and one *M. caprae*. The PCR *pncA*-RFLP had a sensitivity of 100.0% (95%CI: 93.3 -100.0) and a specificity of 100.0% (95%CI: 87.9–100.0) for identifying *M. bovis*. The RD-PCR showed that all *M. bovis* strains had the RD9 and RD4 deleted but presented RD1. RD-PCR also presented high sensitivity and specificity in detecting *M. bovis* strains (100.0%).

**Conclusions:**

PCR *pncA*-RFLP and RD-PCR represent very accurate and rapid tools to identify *M. bovis*. They can be easily implemented in each laboratory due to their low cost and easy use.

## Introduction

Zoonotic Tuberculosis (zTB) is caused principally by *Mycobacterium bovis* and other species of *Mycobacterium tuberculosis* complex (MTBC) e.g; *M. caprae, M. pinnipedii*, *M. microti, M. orygis* 1,2,3,4,5.

The World Health Organization (WHO) estimates 147,000 new human cases in 2016 due to zTB with 12,500 deaths 6. In Tunisia, lymph node TB incidence was increased from 2.3 cases/100,000 inhabitants in 1993 to 18.0 cases/100,000 inhabitants in 2017 and *M. bovis* could be responsible for 78.9% of lymph node TB cases 7. *M. bovis* is intrinsically resistant to pyrazinamide (PZA) due to the mutation C169G of *pncA* gene (codon 57:H57D) 8.

Phenotypic and biochemical tests traditionally used to identify this species are time-consuming and inaccurate^9^. The WHO recommended identifying this species to estimate the burden of zTB in each setting and prescribe an adequate treatment 1. Various methods have been developed for this purpose.

Sequencing based genotyping methods have been used as a reference standard to well differentiate between MTBC species. A set of molecular markers has been used for this aim, such as 16S rRNA, *oxyR, katG, pncA, gyrA, gyrB* and *hsp65* 10–11. However, sequencing-based genotyping methods are expensive and require specific equipment.

At the national reference laboratory (NRL) for mycobacteria in Tunisia, the line probe assay: Genotype MTBC (Hain Lifescience, Germany) is used for molecular identification of *M. bovis* strains, whereas, this method is costly (34 $ for one test)

Herein, two cost-effective PCR approaches are evaluated: a PCR *pncA*-Restriction Fragment Length Polymorphism (RFLP) and a multiplex PCR based on the detection of three Region of Difference (RD9, RD4 and RD1) for the detection of *M. bovis* in lymph nodes cultures, in comparison with the line probe assay: GenoType MTBC assay.

## Materials and methods

### Ethical approval

This study is approved by the ethics committee of A. Mami pneumology hospital, Ariana, Tunisia.

### Clinical specimens, strains identification and phenotypic Drug susceptibility testing (DST)

Two hundred sixty-four lymph nodes samples (n=264) were tested at the NRL for mycobacteria in Tunisia, during 2013- 2015. They were subjected to: acid-fast bacilli smear examination, a culture in liquid medium Mycobacteria Growth Indicator Tube 960 “MGIT960” (BD, USA), a culture in solid medium: Lowenstein Jensen “LJ”, and a molecular diagnosis by GeneXpert MTB/RIF (Cepheid, USA).

MTBC species identification was carried out by SD TB Ag MPT64 Rapid kit (Standard Diagnostics, South Korea), biochemical tests: niacin production, nitrate reductase activity, growth on thiophene-2-carboxylic acid hydrazide and the molecular assay GenoType MTBC.

To study the specificity of evaluated methods, different MTBC species selected from our strains bank: *M. caprae*, *M. bovis, M. bovis* BCG and *M. tuberculosis* H37Rv were included in addition to 7 species of non-tuberculous mycobacteria (NTM): *M. chelonae, M. abscessus, M. kansasii*, *M. intracellulare, M. fortuitum, M. marinum, M. peregrinum*. The NTM were identified by GenoType Mycobacterium CM/AS assay (Hain Lifescience, Germany). The phenotypic DST for first-line drugs was performed on MGIT 960 or LJ. For PZA, it was performed on MGIT 960 PZA kit (BD, USA).

### PCR *pncA*-RFLP and RD-PCR

DNAs were extracted from MGIT 960 cultures. One ml of MGIT was centrifuged at 12.000 rpm for 10 min. The pellets were suspended in 200 µl of Tris EDTA Buffer (10 mMTris-Cl pH 8.0, 1 mM EDTA) and heated at 95°C for 30 min. The suspensions were then centrifuged at 13,000 rpm for 15 min and the supernatants were kept and frozen at -20°C.

The PCR mixture (25µl) for PCR pncA-RFLP method was prepared using 2.5 µl of buffer (10×), 0.1 µl of primers pncA F et R (25µM) 11, 2 µl of dNTP (10 mM), 2.5 µl of MgCl2 (25 mM), 2.5 µl of DNA, 0.15 µl of Bioamtik Taq polymerase (500U) and water. The amplification was performed, according to Huard et al.^11^. The PCR products (664bp) were digested by *BstEII* enzyme (New England, UK).

If 2 bands were obtained (170 bp and 494 bp): a mutation at position 169 of *pncA* is present.

If 3 bands were found (103 bp, 170 bp, and 391 bp): no mutation at position 169 of *pncA* gene.

For the RD-PCR method, the mix (25 µl) was composed of 2.5 µl of buffer (10×), 0.5 µl of primers F, R and int for each region RD1, RD4 and RD9 (25 µM)^12^,^13^, 4 µl of dNTP (10 mM), 1 µl of MgCl2 (50mM), 2.5 µl of DNA, 0.15 µl of Platinum Taq polymerase (Invitrogen, USA) and water.

The amplification was performed, according to Warren et al. 13. The size of the bands, after electrophoresis, allows to deduce the absence (-) or presence (+) of the target regions: RD1+: 146bp; RD1-: 196bp; RD4+: 172bp; RD4-: 268bp; RD9+: 235bp; RD9-: 108bp 13.

The species of MTBC, including *M. bovis*, were identified based on the presence or absence of these 3 RD.

### Data analysis

Sensitivity, Specificity, Positive and Negative Predictive Values (PPV/NPV) were calculated using Open Epi version 3.01 with a confidence interval (CI) of 95%.

## Results

During 2013–2015, lymphadenitis TB was confirmed in 164 cases (62.12%) by microscopy and /or culture and/or GeneXpert MTB/RIF. The culture was positive in 82 cases (50.0%). GenoType MTBC assay showed that TB lymphadenitis was due to *M. bovis* (n=54), *M. tuberculosis* (n=27) and *M. caprae* (n=1).

All *M. bovis* strains were resistant to PZA by MGIT960.

### Molecular identification by PCR pncA-RFLP

**Figure 1:**
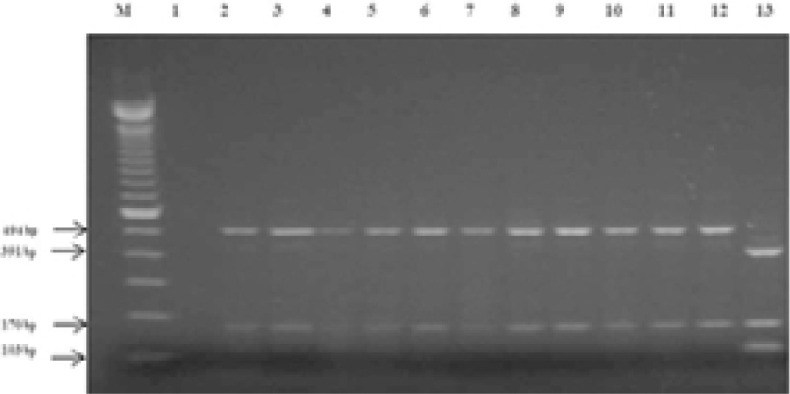
Results of PCR *pncA*-RFLP M: 100bp DNA ladder, 1: *M. kansasii* (NTM: control strain), 2, 3, 4, 5, 6, 7, 8, 9, 10, 11: *M. bovis*, 12:*M. bovis BCG* (control strain), 13: *M. tuberculosis*

PCR *pncA*-RFLP showed that 54 *M. bovis* strains presented 2 bands of 170 bp and 494 bp after digestion by BstEII (Figure 1). It showed that 28 strains presented 3 bands of 103bp, 170bp and 391bp (Figure 1). Twenty-seven were *M. tuberculosis* and one strain was *M. caprae*, according to GenoType *MTBC*.

PCR-RFLP had a sensitivity of 100.0% (95%CI: 93.3 -100.0), a specificity of 100.0 % (95 CI: 87.9–100.0), a PPV of 100.0% (95%CI:93.3 -100.0) and a NPV of 100.0% (95% CI:87.9–100.0) for detecting *M. bovis*.

As regards the control strains: *M. bovis* and *M. bovis* BCG presented 2 bands after the digestion, whereas, *M. tuberculosis* H37Rv and *M. caprae* showed 3 bands.

No amplification of *pncA* was detected for NTM species.

### Molecular identification by Regions of Difference

**Figure 2:**
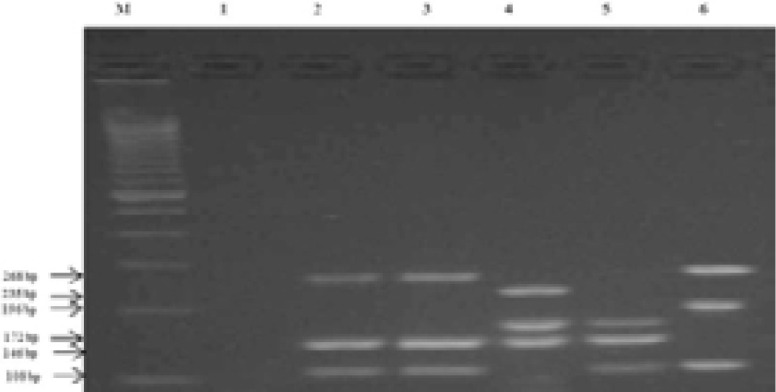
Results of the identification by RD M: 100bp DNA ladder, 1 ***M. kansasii*** (NTM: control strain), 2 and 3 ***M. bovis***: 268bp (RD4-), 146bp (RD1+), 108bp (RD9-), 4 ***M. tuberculosis***: 235bp (RD9+) 172bp, (RD4+), 146 bp (RD1+), 5*M. caprae:*172 bp (RD4+), 146bp (RD1+), 108bp (RD9-), 6 ***M. bovis BCG*** (control strain): 268bp (RD4 -), 196bp (RD1-), 108 bp (RD9-)

All *M. bovis* strains (n=54) had RD9 and RD4 deleted. Our results showed that 27 strains presented the 3 RD targeted (RD9+/RD4+/RD1+) (Figure 2).

These strains belonged to (*M. tuberculosis*/ *M. canettii*) group. The biochemical tests and the GenoType MTBC identify these strains as *M. tuberculosis*. The *M. caprae* strain showed the absence of RD9 and the presence of RD4 and RD1 and was classified in (*M. caprae*/ *M. africanum* /*M. pinnipedii* and *M. microti*) group.

The sensitivity of RD-PCR for identifying *M. bovis* was 100.0% (95%CI: 93.3 -100.0) with a specificity of 100.0% (95% CI: 87.9–100.0).

As concerns the control strains: *M. tuberculosis* H37Rv had the 3 RD studied, *M. caprae* presented only RD9, *M. bovis* had RD9 and RD4 deleted and *M. bovis* BCG had the 3 RD deleted. No amplification was found for the NTM species.

## Discussion

*Mycobacterium bovis* is an important cause of TB in humans. Accurate, rapid identification of this species is required to allow appropriate treatment and set a strategy to monitor the cattle's disease. For this purpose, two cost-effective PCR approaches were evaluated in comparison with the molecular assay: GenoType MTBC.

The molecular identification based on the polymorphism at position 169 of *pncA* presented very high sensitivity and specificity in detecting *M. bovis* strains (100.0%). This method could also represent a rapid tool to detect the natural resistance to PZA. In fact, it is known that three MTBC species are intrinsically resistant to this drug: *M. bovis, M. bovis BCG*, due to the *pncA* C169G substitution and *M. canettii* 14,15.

The allelic variation at *oxyR* position 285 has also been proposed to differentiate *M. bovis* from *M. tuberculosis* but did not distinguish between *BCG* and non-*BCG M. bovis* strains 11,16,17.

In addition, a multiplex PCR was tested to detect the presence or absence of 3RD: RD9, RD4 and RD1^12^,^13^. The *RDs* represent the loss of genetic materials in *M. bovis BCG* compared to *M. tuberculosis* H37Rv genome^11^. All *M. bovis* strains in this study (n=54) had RD9 and RD4 deleted but presented RD1. Consequently, the RD-PCR showed excellent sensitivity and specificity (100.0%) for identifying *M. bovis* isolates.

Compared with the conventional methods, *pncA*-RFLP and RD-PCR represent accurate and fast tools (few hours versus many weeks for biochemical tests) to identify and differentiate *M. bovis* from other MTBC members and NTM species. Furthermore, they have a low cost compared to GenoType MTBC (1.8$ versus 34$ for one test) and do not require expensive equipment and reagents as squencing.

This study had some limitations: first, the two methods were tested using MTBC isolates and were not evaluated directly in lymph nodes samples. Second, mutation *pncA* C169G was also found in *M. bovis* BCG strains 8,14. In addition, some PZA resistant *M. tuberculosis* isolates could display a mutation at this position. Consequently, these strains could be misidentified by *pncA*-RFLP as *M. bovis*.

However, *M. bovis* BCG is rarely isolated from lymph node samples. In addition, a recent study in Tunisia has not reported any mutation at this position in PZA resistant *M. tuberculosis* isolates 18.

Finally, it was shown that some *M. caprae* strains and some *M. tuberculosis* isolates belonging to lineage 3 displayed the RD4 deleted 2,19,20. Despite this finding, RD4 cannot be ruled out until further genomic deletion will be found to well distinguish between these species 19.

## Conclusions

*pncA*-RFLP and RD-PCR represent a rapid, accurate tools to detect *M. bovis* in tuberculosis lymph nodes cultures compared with phenotypic and biochemical tests. They could be implemented easily in each laboratory owing to their easy use and low cost, in comparison with the DNA strip assay: GenoType MTBC and sequencing.

## References

[1] World Health Organization https://www.who.int/tb/areas-of-work/zoonotic-tb/ZoonoticTBfactsheet2017.pdf?ua=11.

[2] Rodríguez S, Bezos J, Romero B, de Juan L, Álvarez J, Castellanos E (2011). Mycobacterium caprae infection in livestock and wildlife, Spain. Emerg Infect Dis.

[3] Silva-Pereira TT, Lkuta CY, Zimpel CK, Camargo NCS, de Souza Filho AF (2019). Genome sequencing of Mycobacterium pinnipedii strains: genetic characterization and evidence of superinfection in a south American sea lion (Otaria flavescens). BMC Genomics.

[4] Emmanuel FX, Seagar AL, Doig C, Rayner A, Claxton P, Laurenson I (2007). Human and animal infections with Mycobacterium microti, Scotland. Emerg Infect Dis.

[5] Duffy SC, Srinivasan SS, Schilling MA, Stuber T, Danchuk SN, Michael JS (2020). Reconsidering Mycobacterium bovis as a proxy for zoonotic Tuberculosis: a molecular epidemiological surveillance study. The lancet Microbe.

[6] World Health Organization (2018). The challenges of preventing bovine Tuberculosis.

[7] Direction des Soins et Santé de Base (2018). Guide de prise en charge de la tuberculose en Tunisie. Ministère de la santé, république Tunisienne.

[8] Scorpio A, Zhang Y (1996). Mutations in pncA, a gene encoding pyrazinamidase/nicotinamidase, cause resistance to the antituberculous drug pyrazinamide in tubercle bacillus. Nature.

[9] Bouakaze C, Keyser C, de Martino SJ, Sougakoff W, Veziris N, Dabernat H (2010). Identification and Geno-Typing of Mycobacterium tuberculosis complex species by use of SNaPshot Minisequencing-based assay. J Clin Microbio.

[10] Brosch R, Gordon SV, Marmiesse M, Brodin P, Buchrieser C, Eiglmeier K (2002). A new evolutionary scenario for the Mycobacterium tuberculosis complex. PNAS.

[11] Huard RC, Lazzarini OLC, Ray Butler W, van Soolingen D, Ho JL (2003). PCR-based method to differentiate the subspecies of the Mycobacterium tuberculosis complex on the basis of genomic deletions. J Clin Microbiol.

[12] Parsons LM, Brosch R, Cole ST, Somoskovi Á, Loder A, Bretzel G (2002). Rapid and Simple Approach for Identification of Mycobacterium tuberculosis Complex Isolates by PCR-Based Genomic Deletion Analysis. J Clin Microbiol.

[13] Warren RM, Gey van Pittius NC, Barnard M, Hesseling A, Engelke E, de Kock M (2006). Differentiation of Mycobacterium tuberculosis complex by PCR amplification of genomic regions of difference. Int J Tuber Lung Dis.

[14] Feuerriegel S, Köser CU, Richter E, Niemann S (2013). Mycobacterium canettii is intrinsically resistant to both pyrazinamide and pyrazinoic acid. J Antimicrob Chemother.

[15] Zhang Y, Mitchison D (2003). The curious characteristics of pyrazinamide: a review. Inter J Tuber Lung Dis.

[16] Teo JWP, Cheng JWS, Jureen R, Lin RTP (2013). Clinical utility of RD1, RD9 and hsp65 based PCR assay for the identification of BCG in vaccinated children. BMC Res Notes.

[17] Sreevatsan S, Escalante P, Pan X, Gillies DA, Siddiqui S, Khalaf CN (1996). Identification of a polymorphic nucleotide in oxyR specific for Mycobacterium bovis. J Clin Microbiol.

[18] Bouzouita I, Cabibbe AM, Trovato A, Draoui H, Ghariani A, Midouni B (2018). Is sequencing better than phenotypic tests for the detection of pyrazinamide resistance?. Inter J Tuber Lung Dis.

[19] Domogalla J, Prodinger WM, Blum H, Krebs S, Gellert S, Müller M (2013). Region of Difference 4 in Alpine Mycobacterium caprae Isolates Indicates three Variants. J Clin Microbiol.

[20] Faksri K, Xia E, Tan JH, Teo YY, Ong RTH (2016). In silico region of difference (RD) analysis of Mycobacterium tuberculosis complex from sequence reads using RD-Analyser. BMC Genomics.

